# Self-Monitoring in Speaking: In Defense of a Comprehension-Based Account

**DOI:** 10.5334/joc.61

**Published:** 2020-09-03

**Authors:** Ardi Roelofs

**Affiliations:** 1 Radboud University, Nijmegen, NL

**Keywords:** Auditory word processing, Executive functions, Language production, Neuropsychology, Response accuracy, Eye movements

## Abstract

Speakers occasionally make speech errors, which may be detected and corrected. According to the comprehension-based account proposed by Levelt, Roelofs, and Meyer (1999) and Roelofs (2004), speakers detect errors by using their speech comprehension system for the monitoring of overt as well as inner speech. According to the production-based account of Nozari, Dell, and Schwartz (2011), speakers may use their comprehension system for external monitoring but error detection in internal monitoring is based on the amount of conflict within the speech production system, assessed by the anterior cingulate cortex (ACC). Here, I address three main arguments of Nozari et al. and Nozari and Novick (2017) against a comprehension-based account of internal monitoring, which concern cross-talk interference between inner and overt speech, a double dissociation between comprehension and self-monitoring ability in patients with aphasia, and a domain-general error-related negativity in the ACC that is allegedly independent of conscious awareness. I argue that none of the arguments are conclusive, and conclude that comprehension-based monitoring remains a viable account of self-monitoring in speaking.

## Introduction

In verbal communication, speakers occasionally make slips of the tongue, which may be detected and corrected. Disagreement exists about how error monitoring is achieved. According to the comprehension-based account proposed by Levelt, Roelofs, and Meyer (1999) and Roelofs (2004), speakers detect errors by using their speech comprehension system. Errors can be detected internally before articulation onset (the internal loop) or in actually produced overt speech (the external loop). According to the production-based account of self-monitoring proposed by Nozari, Dell, and Schwartz (2011), speakers may use their speech comprehension system to monitor for errors in overt speech (“there is no doubt that monitoring overt speech through comprehension occurs”, p. 5). However, error detection in internal monitoring is assumed to be based on the amount of conflict within the speech production system, taken to be assessed by the anterior cingulate cortex (ACC) following Yeung, Botvinick, and Cohen (2004). Nozari et al. assume that speakers “are capable of monitoring their inner speech when they are not speaking aloud” (p. 3). Conflict monitoring is proposed as an account of internal monitoring when speaking aloud. It is taken to be “the default mechanism of error-detection in language production” (p. 2).

Whereas the comprehension-based account assumes a single mechanism for both internal and external monitoring, the production-based account assumes one mechanism (i.e., conflict detection, “the default mechanism”) for internal monitoring and another mechanism (i.e., the comprehension system) for external monitoring and for monitoring inner speech when not speaking aloud. If a self-monitoring mechanism exists for the monitoring of overt speech, it is plausible to assume that the same mechanism is also used for the monitoring of inner speech, as the comprehension-based account assumes.

Nozari et al. (2011) and Nozari and Novick (2017) give three main arguments against the comprehension-based account of internal monitoring. The arguments concern (1) the theoretical question of how a speaker can distinguish between inner and overt speech, and prevent cross-talk interference between them (Vigliocco & Hartsuiker, 2002), (2) a double dissociation between comprehension and self-monitoring ability in patients with aphasia (Nozari et al.), and (3) an error-related negativity (ERN) in the event-related brain potential that is domain-general, supposedly independent of conscious awareness of errors, and arises in the ACC (Nozari & Novick).

The aim of the present article is to show that these three arguments against a comprehension-based account of internal self-monitoring are not conclusive. I first describe the comprehension-based and production-based accounts in more detail. In the remainder, I focus on monitoring for errors in single word production, although the comprehension-based account also addresses the self-monitoring of phrases and sentences and aspects like appropriateness and speed (Levelt, 1983, 1989). First, I argue that the activation-verification account proposed by Levelt et al. (1999) takes care of cross-talk interference between inner and overt speech. Second, I make clear that, on the comprehension-based account proposed by Roelofs (2004, 2005), self-monitoring uses the speech comprehension system but additionally involves a comparison process. This process is under executive control, associated with the ACC and other frontal areas. Moreover, the comprehension system is directly fed by the production system in self-monitoring but not in comprehending others. This makes self-monitoring and comprehension of others differently sensitive to damage, which may explain the double dissociation in patients with aphasia. Third, I explain why a domain-general ERN is compatible with comprehension-based monitoring, challenge the claim that the ERN is independent of error awareness, and review empirical evidence against ACC conflict monitoring. I argue instead for a role of the ACC in exerting and adjusting executive control. My conclusion is that in the light of the available evidence, the comprehension-based theory remains a viable account of internal self-monitoring in speaking.

## Comprehension-Based Versus Production-Based Internal Monitoring

Comprehension-based and production-based accounts differ in the proposed mechanism for internal monitoring. Evidence for internal monitoring, in addition to external monitoring, comes from a number of sources, including the following ones. First, evidence for internal monitoring comes from the speed of speech interruption after an error, which may happen with a delay as short as 100–150 ms (Blackmer & Mitton, 1991). Such delays are too short for error detection via the external loop based on auditory perception. Second, there is evidence that speakers may still detect errors when their external loop is blocked by masking overt speech through noise. Lackner and Tuller (1979) elicited speech errors with or without masking noise. They observed that errors were detected even when overt speech was masked. Error detection was faster with than without masking noise. This suggests that with masking noise, errors were detected through internal monitoring, which led to faster error detection than through external monitoring. Third, error biases suggest that speakers internally monitor their speech. Motley, Camden, and Baars (1982) used a procedure to elicit errors involving taboo words while measuring the galvanic skin response of speakers. They observed that participants made fewer phoneme exchanges when the error would lead to taboo words (e.g., “shit head” for “hit shed”) as compared to normal exchanges (e.g., “bad mack” for “mad back”). However, a larger galvanic skin response was observed in the taboo condition than in the control condition. This suggests that speakers detected the taboo word outcomes through internal monitoring and suppressed the words before speech onset. Fourth, there is evidence that error biases are differentially present in inner and overt speech. Errors in overt speech show a lexical bias (i.e., errors tend to result in words rather than pseudowords) and a phonemic similarity bias (i.e., exchanging phonemes tend to share phonological features). Oppenheim and Dell (2008) examined these effects in inner and overt speech using a tongue-twister recitation task. They observed that lexical error bias was present in both inner and overt speech, but the phonemic similarity bias was present only in overt speech. This indicates that errors may be detected in inner speech, which seems impoverished in terms of phonological features.

### Comprehension-based monitoring

According to the comprehension-based account proposed by Levelt (1983, 1989), Levelt et al. (1999), and Roelofs (2004), there are two self-monitoring loops, an internal and an external one, both operating via the speech comprehension system (see also Indefrey, 2011; Indefrey & Levelt, 2004). Self-monitoring is under executive control (e.g., Roelofs & Hagoort, 2002), which is one component of the human attention system, associated with the ACC and other frontal areas (e.g., Posner, 2012; see Roelofs & Piai, 2011, for a review of the attention demands of spoken word production). Monitoring implies some degree of conscious awareness. As Levelt (1989) put it: “Message construction is controlled processing, and so is monitoring; self-corrections are hardly ever made without a touch of awareness. The speaker can *attend* to his own internal or overt speech” (p. 21, italics original). The external loop involves listening to self-produced overt speech, whereas the internal loop involves monitoring the speech plan by feeding an incrementally generated phonological word back into the speech comprehension system. A phonological word representation specifies the syllables and, for polysyllabic words, the stress pattern across syllables. In Roelofs (2004, 2005), I proposed that errors are detected by comparing representations selected for production with representations selected in comprehension.

**Figure 1:**
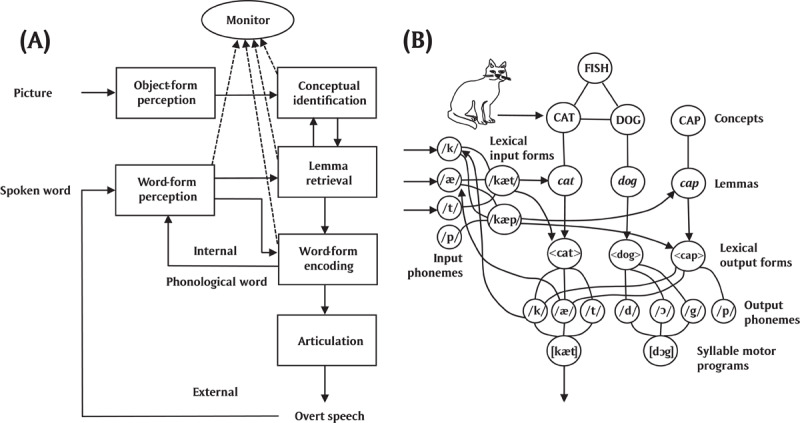
Illustration of comprehension-based self-monitoring in WEAVER++. **(A)** In naming a picture, the phonological word representation of the picture name is fed into the speech comprehension system (the internal loop), which also processes the overtly articulated picture name (the external loop). The monitor compares selected production and comprehension representations. **(B)** Network representing the word *cat*, whereby output phonemes activate input phonemes, which serves the internal monitoring loop.

Figure 1 illustrates the external and internal loops in the WEAVER++ model (Levelt et al., 1999; Roelofs, 2004, 2005, 2014). Figure 1A shows that the naming of a picture involves conceptually identifying the pictured object based on its perceived form (e.g., a cat), retrieving the corresponding lemma, also called lexical selection (e.g., the lemma of the word *cat*), the encoding of the word form (involving morphological, phonological, and phonetic encoding), and finally articulation. The figure shows that a phonological word representation of the picture name is fed into the speech comprehension system (the internal loop), which also processes the overtly articulated picture name (the external loop). The monitor compares selected production and comprehension representations (Roelofs, 2004, 2005). Figure 1B shows the lexical network assumed by the WEAVER++ model representing the word *cat*, whereby output phonemes activate input phonemes, which underpins the internal monitoring loop.

The WEAVER++ model makes a distinction between declarative (i.e., structured-symbolic associative memory) and procedural (i.e., condition-action rule) aspects of spoken word planning. The associative network is accessed by spreading activation while condition-action rules select nodes among the activated lexical information depending on the task demands specified in working memory (e.g., to name a picture). Activation spreads continuously from level to level, whereby each node sends a proportion of its activation to connected nodes. The lexical network illustrated in Figure 1B consists of lexical concepts (e.g., CAT), lemmas (e.g., *cat*), output lexical forms or morphemes (e.g., <cat>), output phonemes (e.g., /k/, /æ/, and /t/), and syllable motor programs (e.g., [kæt]). Lemmas specify the syntactic properties of words (not shown in Figure 1B), crucial for the production of phrases and sentences. Internal monitoring is enabled by the connections from output to input phonemes, which allow a constructed phonological word representation to be processed by the speech comprehension system for monitoring purposes.

The existence of internal and external monitoring loops raises the question of what their relative contribution is in detecting errors. Hartsuiker, Kolk, and Martensen (2005) presented a probabilistic model that used empirical data on error detection to estimate the relative contributions of the two loops. Based on model fits, they conclude that more errors are detected by the internal than the external loop, except in case of phonological errors, where the contributions of the loops seem to be equal. However, patients with Broca’s aphasia seem to rely on the internal loop only (with covert repairs contributing to their nonfluent speech). Hartsuiker et al. argue that the relative contribution of the two loops is determined by selective attention (see also Roelofs, 2004). Giving more attention to the internal than the external loop will prevent errors and avoids the need to repair the overt errors after they are made.

The comprehension-based account of self-monitoring that I proposed in Roelofs (2004, 2005) falls into the general class of feedback-based monitoring models, which includes the DIVA model of Guenther (2016), the HSFC model of Hickok (2012), and the model of Kröger, Crawford, Bekolay, and Eliasmith (2016), among others. Levelt (1983, 1989) proposed comprehension-based monitoring but did not assume a comparison process at all levels. Hickok assumes monitoring at syllable and phoneme levels based on internal and external feedback. In my proposal, self-monitoring via the speech comprehension system requires cognitive operations to detect discrepancies between selections made in production and comprehension. For example, lexical selection errors may be detected by verifying whether the lemma of the recognized word corresponds to the lexical concept selected in production. Phoneme selection errors may be detected by verifying whether the recognized phonemes correspond to the selected lexical form in production. WEAVER++ implements such verification operations by means of condition-action production rules. Note that relevant production and comprehension representations are close to each other in the network. Different sets of production rules may perform the monitoring operations for the different planning levels. A related account has been advanced by Kröger et al., who proposed a biologically inspired model of comprehension-based internal monitoring. In their model, about 500,000 spiking neurons implemented structured symbolic representations and condition-action production rules, which achieved semantic and phonological error detection in computer simulations of picture naming.

### Production-based monitoring

According to the production-based account of internal monitoring proposed by Nozari et al. (2011), error detection is based on the amount of conflict within the speech production system, taken to be assessed by the ACC following Yeung et al. (2004). Nozari et al. assume that speakers use conflict as an error signal in internal monitoring and that they may use their speech comprehension system to monitor for errors in overt speech.

The model of Nozari et al. (2011) holds that in naming a picture, the semantic features corresponding to the pictured object become activated (e.g., the features FELINE, MEOWING, and ANIMAL for cat), followed by a spread of activation to associated word nodes (e.g., *cat*) and phoneme nodes (e.g., /k/). After a constant number of time steps (i.e., eight), the highest activated word node is selected (e.g., *cat*) and the amount of conflict is determined. Conflict concerns the difference in activation between the two most highly activated nodes (e.g., *cat* and *dog*). After another constant number of time steps (i.e., eight), the highest activated onset, vowel, and coda phonemes are selected (e.g., /k/, /æ/, and /t/) and the amount of conflict is determined. A small activation difference means high conflict. If conflict at the word or phoneme level exceeds a criterion, the conflict monitor assumes a selection error and rejects the overt naming response that is made.

## The Cross-Talk Problem

A comprehension-based monitoring account predicts perception-specific effects, like an effect of the perceptual uniqueness point of words, in the monitoring of inner speech. The uniqueness point is the phoneme at which the word form diverges from all other words in the language, going from the beginning of the word to its end. The position of the uniqueness point in words influences the speed of spoken word recognition (e.g., Marslen-Wilson, 1990). Özdemir, Roelofs, and Levelt (2007) conducted a study testing for effects of the perceptual uniqueness point using internal phoneme monitoring and overt picture naming tasks. In one block of trials, participants were presented with pictured objects and they indicated by pressing a button whether the picture name contained a pre-specified target phoneme (e.g., picture of a cat, target phoneme /t/). The position of the target phonemes varied relative to the uniqueness point of the picture names. The prediction was that internal monitoring latencies should depend on the distance of the phoneme from the uniqueness point of the picture name (cf. Marslen-Wilson). Moreover, the linear position of the target phoneme within the picture names varied to replicate the results of Wheeldon and Levelt (1995) and Wheeldon and Morgan (2002), who observed that phonemes at the beginning of a word are reported faster than medial and final phonemes in internal monitoring. In another block of trials, participants were asked to overtly name the pictured objects. According to the comprehension-based account, effects of uniqueness point and linear position should be present in the latencies of internal phoneme monitoring but not of picture naming. This is what Özdemir et al. observed, supporting comprehension-based monitoring of inner speech.

Nozari et al. (2011) acknowledge that speakers “are capable of monitoring their inner speech when they are not speaking aloud” (p. 3). However, they followed Vigliocco and Hartsuiker (2002) in claiming that comprehension-based monitoring during overt speech suffers from a cross-talk problem. In discussing Levelt et al. (1999), Vigliocco and Hartsuiker stated that in processing inner and overt speech:

“The comprehension system needs to deal with two phonetic codes that represent the same message and that are only slightly asynchronous. How can the comprehension system distinguish between the two codes, and how does it prevent interference between them? If these two codes are processed with a slight delay, why do we not experience the consequences of this delay (e.g., “echoes”)” (p. 466).

Nozari et al. argue that “listening to both would be similar to constantly listening to an echo of your voice, which would make comprehension difficult.” (p. 3). It should be noted however that, different from what Vigliocco and Hartsuiker suggest, comprehension-based monitoring as proposed by Levelt et al. does not concern internal monitoring of phonetic codes but of phonological representations, which lack phonetic detail. Why then should speakers experience an echo? The echo problem does not seem to apply to the theory of Levelt et al. Vigliocco and Hartsuiker emphasize the cross-talk interference aspect: How to distinguish between the two codes and how to prevent interference between them?

As a solution to the problem of cross-talk interference between internal and external speech, Vigliocco and Hartsuiker (2002) proposed that speakers cannot monitor inner speech during the production of overt speech. Similarly, Huettig and Hartsuiker (2010) argued that “speakers can only ‘listen’ to internal speech when performing a silent task (like Özdemir et al.’s [2007] phoneme monitoring task), but not when speaking out loud” (p. 350). However, Wheeldon and Levelt (1995) reported an effect of linear position of the target phoneme on internal phoneme monitoring latencies that was independent of whether or not participants spoke aloud. In particular, using a word translation task, Wheeldon and Levelt asked participants to perform internal phoneme monitoring with or without a concurrent articulation task, which consisted of overtly counting from one onwards (i.e., the production of something unrelated). Overt production of numbers did not affect the pattern of monitoring results. This shows that speakers can deal with concurrent internal and external speech, even when they represent different content (here, the translated word and numbers). In summary, the uniqueness point effect obtained by Özdemir et al. suggests that internal phoneme monitoring is accomplished by using the speech comprehension system, and the findings of Wheeldon and Levelt suggest that internal phoneme monitoring may be done while speaking aloud. Thus, unlike what Nozari et al. (2011), Vigliocco and Hartsuiker, and Huettig and Hartsuiker claim, the empirical evidence suggests that speakers are capable of monitoring their inner speech when they are speaking aloud.

### Revisiting the evidence from eye movements

To test whether internal monitoring happens in a task involving overt speech production, Huettig and Hartsuiker (2010) examined the influence of inner and overt speech on eye movements. Earlier studies have shown that when participants hear a word (e.g., *beaker*) while perceiving a visual display with several written words that are spatially separated, they tend to fixate more often on phonologically related words (e.g., *beaver*) than on unrelated words. Huettig and Hartsuiker asked participants to name pictures in displays with phonologically related and unrelated written words. They observed that the tendency to fixate more on phonologically related than unrelated words occurred after rather than before speech onset in picture naming. According to Huettig and Hartsuiker, this provides evidence that speakers do not monitor inner speech in an overt production task.

The problem with this conclusion of Huettig and Hartsuiker (2010) is that comprehension-based monitoring does not imply that eye movements are driven by inner speech. Elsewhere, I argued that whether eye movements are initiated before or after speech onset in picture naming depends on the task demands (Roelofs, 2007, 2008). In eye-tracking experiments, participants were presented with pictures displayed on the left side of a computer screen and left- or right-pointing arrows simultaneously displayed on the right side of the screen. Participants vocally named the picture and shifted their gaze to the arrow to manually indicate its direction. The instruction was to give the vocal and manual responses as quickly as possible without making mistakes. Different from what Huettig and Hartsuiker observed, the eye movement from the picture to the arrow was already initiated before overt speech onset. Note that the words in the displays of Huettig and Hartsuiker were not relevant for the task at hand, which was simply to name the picture. Thus, for their participants there was no need to fixate any of the words while planning the picture name. In contrast, for the participants in my own experiments it made sense to shift gaze before overt speech onset, because this would lead to a faster response to the arrow. Indeed, when early gaze shifts were made unnecessary by presenting the arrow a second after picture onset, the gaze shift was initiated after overt speech onset, corresponding to what Huettig and Hartsuiker observed. This suggests that in naming pictures, speakers shift their gaze away from the picture after overt speech onset if the task situation allows this. Gaze shifts are not determined by the monitoring of inner speech but by task demands, and thus provide no evidence on whether inner speech is monitored or not during picture naming.

To conclude, the eye movement data are neutral with respect to the issue of whether or not internal monitoring is comprehension-based. The findings of Huettig and Hartsuiker (2010) do not exclude that speakers monitor their inner speech in an overt production task. Thus, the question remains how speakers solve the cross-talk problem.

### Dealing with the cross-talk problem

To deal with cross-talk between speaking and listening, the comprehension-based monitoring model of Levelt et al. (1999) and Roelofs (2004, 2005) assumes an activation-verification mechanism, which creates processing threads that allow speakers to distinguish between production, internal monitoring, and external monitoring streams (cf. Salvucci & Taatgen, 2008, 2011). In planning the name of a picture, a concept is selected for a selected percept, a lemma is selected for a selected concept, one or more morphemes are selected for a selected lemma, phonemes are selected for the selected morphemes and syllabified to create a phonological word representation, and syllable motor programs are selected for the syllables in the phonological word. Thus, selection of nodes from a spreading activation network creates a thread of selected nodes representing the planned word. Similarly, feeding the constructed phonological word representation into the comprehension system for internal monitoring may yield a thread of selected nodes representing the internally perceived word, and hearing self-produced overt speech may yield a thread of selected nodes in the comprehension system representing the externally perceived word. If the internally and externally perceived words are represented by different processing threads, the comprehension system can distinguish between them and prevent interference. Elsewhere (Roelofs, 1997), I showed the utility of an activation-verification mechanism to account for the latencies of correct picture naming responses while concurrently hearing spoken distractor words creating cross-talk interference.

To conclude, empirical evidence suggests that speakers are capable of monitoring their inner speech when they are speaking aloud. The fact that eye movements are driven by overt speech under some circumstances is neutral with respect to the issue of whether internal monitoring is comprehension-based. Moreover, cross-talk interference is taken care of by the activation-verification account of Levelt et al. (1999).

## A Double Dissociation in Aphasia

An old argument against comprehension-based monitoring is the evidence for a double dissociation between comprehension and self-monitoring ability in persons with aphasia (e.g., Nickels & Howard, 1995). The argument is that if speech comprehension is poor due to brain damage, then self-monitoring also has to be poor because it is done using the impaired speech comprehension system. It should be noted, however, that in agreement with the comprehension-based monitoring account, patients with Wernicke’s aphasia are reported to be less aware of their language deficits than patients with Broca’s aphasia (e.g., Dronkers & Baldo, 2009). This has been attributed to the comprehension deficit in Wernicke’s aphasia, which is assumed to be extended to the monitoring of speech production. Different from Wernicke’s aphasia, comprehension is spared in conduction aphasia. In agreement with comprehension-based monitoring, patients with conduction aphasia are aware of their errors and characteristically make multiple attempts to repair them, called “conduite d’approche”. However, in contrast to these observations on error awareness in classic aphasia types, Nickels and Howard observed that measures of speech comprehension and self-monitoring ability were not correlated in a group of persons with aphasia (but see Roelofs, 2005). Nozari et al. (2011) made the same observation. These observations challenge a comprehension-based account, according to Nozari et al. It should be mentioned that Dean, Della Sala, Beschin, and Cocchini (2017), using different general methods (from definitions, through coding, to analysis), did observe a correlation between self-monitoring and speech comprehension ability in a group of 48 patients with post-stroke aphasia.

According to the comprehension-based account that I proposed in Roelofs (2004, 2005), self-monitoring uses the speech comprehension system but also involves a comparison of comprehension and production representations. Consequently, if brain damage spares speech comprehension but impairs the comparison process, then poor self-monitoring may occur in the context of good comprehension. For example, Marshall, Robson, Pring, and Chiat (1998) report on a patient with poor self-monitoring but good speech comprehension. Therapy improved monitoring by the patient but not his naming, which Marshall et al. explained by assuming that “therapy strengthened the semantic representations of the treated items. As a result, with these items, he was able to compare his production to primed nodes at the semantic level and hence achieve greater error awareness” (p. 104). This suggests that self-monitoring involves abilities that go beyond speech production and comprehension per se. Also, if speech comprehension is poor because brain damage has impaired perception of the external speech signal while sparing the internal loop, then good self-monitoring may occur in the context of poor speech comprehension. For example, Marshall, Rappaport, and Garcia-Bunuel (1985) report on a patient with severe auditory agnosia but preserved self-monitoring ability. She demonstrated poor performance on identifying human nonlinguistic sounds (e.g., kissing), environmental sounds (e.g., typing), animal sounds (e.g., cow mooing), and familiar melodies. However, she performed rather well on tasks tapping into inner speech, such as picking out visual stimuli whose names rhymed. Thus, good comprehension is neither necessary nor sufficient for good self-monitoring. Double dissociation may occur under a comprehension-based monitoring account.

A further complication for the claim that comprehension should predict self-monitoring is that, on the comprehension-based account, the comprehension system is directly fed by the production system in self-monitoring but not in comprehending others. That is, in comprehending the speech of others, the comprehension success depends on how well the patient is able to process the external speech signal, which drives processing from acoustic features via phonemes and lexical items to concepts. In contrast, in self-monitoring via the speech comprehension system, the concepts, lexical items, and phonemes for production directly activate the corresponding representations in the comprehension system (see Figure 1B). As a consequence, self-monitoring may be less sensitive than comprehending others to damage of the comprehension system. Thus, good self-monitoring may be observed in the context of poor comprehension.

To conclude, the comprehension-based monitoring account that I proposed in Roelofs (2004, 2005) assumes that self-monitoring uses the speech comprehension system but also involves a comparison process. Moreover, the comprehension system is directly fed by the production system in self-monitoring but not in comprehending others. As a consequence, a double dissociation between comprehension and self-monitoring ability may occur in patients with aphasia, contrary to what Nozari et al. (2011) claim about comprehension-based monitoring.

### Response rejection by patients

In their model, Nozari et al. (2011) assume that brain damage may reduce the strength of the connections between semantic feature and word nodes (i.e., the semantic weights) or between word and phoneme nodes (i.e., the phonological weights). Reduced connection strength diminishes the utility of conflict as an index of error likelihood. Consequently, patients with low semantic weights will have a low proportion of detected semantic errors in picture naming, and patients with low phonological weights will have a low proportion of detected phoneme errors, which corresponds to what Nozari et al. empirically observed. Thus, production parameters in their model (i.e., connection weights) predict monitoring performance, supporting production-based error-monitoring.

However, there is an issue concerning the empirical evidence reported by Nozari et al. (2011). An error was taken to be detected if a patient attempted to repair the naming response (e.g., “dog … cat”) or rejected the response (e.g., “dog … No”). Yet, it is unclear whether the patients used internal or external monitoring to detect an error. The examples given by Nozari et al. suggest that they corrected or rejected the response after overtly making an error in their naming response, which suggests that external monitoring was involved. However, the conflict-monitoring account of Nozari et al. is about internal monitoring, not about external monitoring, for which they assume that the speech comprehension system may be used. In short, Nozari et al. stipulate that the patients detected all errors through internal monitoring, but they did not provide evidence for that.

To conclude, although several researchers take a double dissociation between comprehension and self-monitoring ability in aphasia to be evidence against comprehension-based monitoring, good comprehension is in fact neither necessary nor sufficient for good self-monitoring under comprehension-based monitoring. Moreover, the production-based conflict monitoring model of Nozari et al. (2011) is about internal monitoring, but it is unclear whether the patient data reflect internal or external monitoring. Furthermore, whereas the patient data of Nozari et al. show no correlation between self-monitoring and comprehension performance, the data of Dean et al. (2017) do show a correlation. Given this empirical state of affairs, strong conclusions cannot be drawn.

## Domain-Generality of the ERN and Its Relation to Error Awareness

The ERN is a negative deflection in the event-related brain potential that occurs around the time of error response onset in both linguistic and nonlinguistic tasks, indicating that it is domain-general. Much evidence suggests that the ERN is generated in the ACC. According to Nozari and Novick (2017), the domain-generality of the ERN challenges comprehension-based monitoring: “It is unlikely that language comprehension is used to detect nonlinguistic errors” (p. 404).

However, contrary to what Nozari and Novick (2017) suggest, comprehension-based monitoring does not imply that the ERN is generated within the speech comprehension system. According to a prominent account of the ERN proposed by Holroyd and Coles (2002), a distinction should be made between a ‘critic’ and a ‘controller’, which entails a distinction between a system that does the monitoring and a system that actually controls the behavior and implements corrections. Holroyd and Coles assume that in reinforcement learning, the basal ganglia have the role of critic and controllers are implemented throughout the brain. In their view, the ACC acts as a ‘control filter’ deciding which action is actually performed. If the critic has detected an error, a learning signal (assumed to be carried by a reduction in dopaminergic activity) is sent to the ACC, where the ERN is generated (assumed to reflect a dopaminergic disinhibition of the apical dendrites of ACC neurons). The learning signal is used by the ACC to modify performance on the task at hand. In this view, the ERN is domain-general but the critic and controllers may be domain-specific. Similarly, in comprehension-based monitoring in speaking, the monitor (critic) may compare production and comprehension representations (cf. Figure 1), and if an error is detected, send an error signal to the ACC, where then an ERN is generated. The error signal is used by the ACC to modify performance of the language production system (controller). In this view, the ACC exerts and adjusts control rather than monitors for conflict.

Thus, different from what Nozari and Novick (2017) maintain, the domain-generality of the ERN does not imply that, under comprehension-based monitoring, the language comprehension system is used to detect nonlinguistic errors. Instead, if during comprehension-based monitoring a linguistic error is detected, this is signaled to the ACC, where consequently an ERN is generated. Similarly, in nonlinguistic domains, a monitor may use nonlinguistic information to detect an error and signal this to the ACC, where the ERN is generated (e.g., Coles, Scheffers, & Holroyd, 2001). Thus, errors in both linguistic and nonlinguistic tasks may yield an ERN in the ACC, making the signal domain-general, while the monitors detecting the errors may use domain-specific information.

According to Nozari and Novick (2017), the “ERN is independent of conscious awareness of errors, which is hard to reconcile with a comprehension-based monitor” (p. 404). However, based on a careful and extensive review of the literature on error awareness and the ERN, Wessel (2012) concluded that:

“It is not possible to uphold the statement that the amplitude of the ERN is unrelated to subjective awareness. On the contrary: while there are many studies that demonstrate enlarged ERN amplitudes with respect to subjective error awareness with a low enough type-1 error probability to warrant rejection of the null-hypothesis …., there are few, if any, studies that have sufficiently low type-2 error probability to warrant an acceptance of that null hypothesis” (pp. 9–10).

Thus, contrary to what Nozari and Novick maintain, extant evidence suggests that the ERN is related to conscious awareness of errors.

To conclude, Nozari and Novick (2017) argued that comprehension-based monitoring is challenged by the domain-generality of the ERN and its independence of conscious awareness. However, the domain-generality of the ERN is compatible with comprehension-based monitoring. Errors in both linguistic and nonlinguistic tasks may yield an ERN in the ACC, making the signal domain-general, while the monitors detecting the errors use domain-specific information. Moreover, different from what Nozari and Novick suggest, the available evidence seems to indicate that the ERN is related to conscious awareness of errors.

## Evidence Against Conflict Monitoring by the ACC

Nozari et al. (2011) stated that their account “borrows from studies of forced-choice-response tasks the notion that error detection is accomplished by monitoring response conflict” (p. 1), thereby referring to the work of Botvinick et al. (2001) and Yeung et al. (2004). Following them, Nozari et al. assume that conflict monitoring in speech production is done “most likely” (p. 9) by the ACC, where the ERN arises. Botvinick et al. proposed the conflict monitoring account of ACC function based on functional magnetic resonance imaging (fMRI) findings obtained with flanker and color-word Stroop tasks. To the extent that ACC conflict monitoring is empirically supported, the proposal of Nozari et al. gains credibility. However, several studies have provided evidence against conflict monitoring by the ACC.

In a seminal study by Botvinick and colleagues (discussed by Botvinick et al., 2001), participants were presented with left- or right-pointing arrows flanked by two congruent or incongruent arrows on each side (e.g., <<<<< or <<><<). They indicated the direction of the central arrow by pressing a left or right button. Reaction time was longer on incongruent than on congruent trials, called the flanker effect. Moreover, the flanker effect was smaller following an incongruent than a congruent trial, called the Gratton effect (Gratton, Coles, & Donchin, 1992). According to the conflict monitoring account, the detection of response conflict on an incongruent trial leads to a decrease of the attentional width on the next trial (i.e., a greater focus on the target arrow), so that the flankers have less influence. As a consequence, the flanker effect in reaction times is reduced. Moreover, ACC activity on incongruent trials was less following incongruent trials than following congruent trials, which was taken as evidence for ACC conflict monitoring. Later studies by Botvinick and colleagues were basically variations on this theme.

A problem with the evidence put forward in favor of the ACC conflict monitoring account is that it is unclear whether the change in attentional width underlying the Gratton effect is driven by previous-incongruent trials (as the conflict monitoring account assumes) or by previous-congruent trials. The latter receives support from the original study of Gratton et al. (1992), where in one of the experiments symbolic cues were used to indicate whether the upcoming stimulus was congruent with 75%, 50%, or 25% probability (in the latter case, the cue is 75%-incongruent predicting). Gratton et al. observed that the flanker effect in reaction times and ERPs was largest following 75%-congruent predicting cues, whereas the magnitude of the flanker effect did not differ between the 50% and 25% cues. This suggests that the control adjustment is driven by expected congruency rather than response conflict. Lamers and Roelofs (2011) replicated this finding of Gratton et al. for reaction times using cues in the color-word Stroop task with vocal responding. Moreover, they conducted other experiments without cues but with neutral trials in addition to incongruent and congruent trials. They observed that the flanker effect in reaction times is largest on post-congruent trials and does not differ between post-incongruent and post-neutral trials, challenging conflict monitoring. This pattern of effects was obtained both for the flanker task and for the Stroop task, and both for manual and vocal responding. Shitova (2018) replicated this pattern of effects for word and phrase production using a picture-word interference paradigm, also challenging conflict monitoring. Using only incongruent and congruent distractor words in picture naming, Freund, Gordon, and Nozari (2016) obtained a Gratton effect in reaction times, which they attributed to conflict monitoring. However, the findings for incongruent, congruent, and neutral distractors obtained by Shitova indicate that the Gratton effect in picture naming is driven by congruent rather than incongruent words. To summarize, the evidence from Gratton et al., Lamers and Roelofs, and Shitova suggests that the Gratton effect is driven by expected congruency rather than response conflict.

Moreover, in an fMRI experiment, Roelofs, Van Turennout, and Coles (2006) observed that ACC activity is larger on incongruent than on neutral trials, but also larger on neutral than on congruent trials, in the absence of response conflict (different from what conflict monitoring predicts, see Figure 1 of Botvinick et al., 2001). Also, Aarts, Roelofs, and Van Turennout (2008) observed that ACC activity is larger in response to incongruent- and congruent-predicting cues than to neutral cues, well before the stimulus is presented and response conflict occurs. These findings suggest that the ACC is not monitoring for conflict (otherwise activity on neutral and congruent trials should not have differed, and the ACC should not have been active in response to congruent-predicting cues). Rather, the evidence suggests that the ACC is involved in exerting and adjusting executive control (cf. Holroyd & Coles, 2002; Roelofs, 2003). Roelofs et al. and Lamers and Roelofs presented the results of computer simulations using the WEAVER++ model showing the utility of such an executive control account of ACC function (see also Roelofs & Hagoort, 2002).

The ACC conflict monitoring account has also been applied to N2 and ERN effects in electrophysiological studies with flanker tasks by Yeung et al. (2004). They claim that the detection of conflict by the ACC is reflected in the N2 during the planning of a response and in the ERN after response onset. However, Burle, Roger, Allain, Vidal, and Hasbroucq (2008) observed that the ERN and the amount of conflict in manual EMG are inversely related. Similarly, Zheng, Roelofs, Farquhar, and Lemhöfer (2018) observed an inverse relation between the amount of conflict and the ERN in bilingual picture naming. Also, Shao, Roelofs, Acheson, and Meyer (2014) observed that in picture naming, the magnitude of N2 and reaction time effects are inversely related. These findings concerning the N2 and ERN are exactly opposite to what the ACC conflict monitoring account would predict.

To conclude, following proponents of the conflict monitoring account (Botvinick et al., 2001; Yeung et al., 2004), Nozari et al. (2011) assume that conflict monitoring in speech production is (“most likely”) done by the ACC. However, evidence against ACC conflict monitoring has been accumulating, both in nonlinguistic and linguistic domains.

In an fMRI study, Gauvin, De Baene, Brass, and Hartsuiker (2016) had participants produce tongue twisters or listen to tongue twisters spoken by someone else. In the production condition, the participants heard white noise to prevent the perception of their overt speech, so that internal monitoring was tested. After production or perception, the participants had to indicate by button press whether the produced or heard tongue twister contained an error. Gauvin et al. observed that errors in the production and perception conditions activated the ACC and other frontal areas. Superior temporal cortex, which is generally taken to underlie speech perception, was generally more active in the perception than in the production condition, and showed a complicated pattern of activations and de-activations in response to errors. According to Gauvin et al., these findings provide evidence in favor of production-based conflict monitoring and against comprehension-based monitoring.

However, as indicated earlier, according to the comprehension-based account that I proposed in Roelofs (2004, 2005), self-monitoring uses the speech comprehension system but also involves a comparison process, which is under executive control. The activation of the ACC and other frontal areas is expected if executive control is involved and the ACC receives error signals (e.g., Piai, Roelofs, Acheson, & Takashima, 2013; Piai, Roelofs, Jensen, Schoffelen, & Bonnefond, 2014; Roelofs, 2003, 2014; Roelofs & Hagoort, 2002). The tongue twisters were designed to be difficult to phonologically encode and pronounce properly, so monitoring will concern form rather than meaning, engaging superior temporal cortex (e.g., Indefrey, 2011; Indefrey & Levelt, 2004; Indefrey & Cutler, 2004). Moreover, given that the perception system is activated differently in production than in listening to others, direct comparison of activity between production and perception conditions in superior temporal cortex is expected to yield a complex pattern of results, especially if error-related activity is examined. This corresponds to what Gauvin et al. (2016) observed.

To conclude, errors in production and perception activate the ACC and other frontal areas as well as superior temporal cortex, albeit in a complicated way. These findings are compatible with comprehension-based monitoring, different from what Gauvin et al. (2016) claim.

## Summary and Conclusions

I addressed three main arguments of Nozari and colleagues (2011; Nozari & Novick, 2017) against a comprehension-based account of internal monitoring in speech production. The arguments concern (1) the theoretical question of how to distinguish between inner and overt speech and how to prevent cross-talk interference between them, (2) a double dissociation between comprehension and self-monitoring ability in patients with aphasia, and (3) an ERN in the event-related brain potential that is domain-general, supposedly independent of conscious awareness of errors, and arising in the ACC. I showed that the cross-talk problem is dealt with by the activation-verification account proposed by Levelt et al. (1999). Moreover, I made clear that, on the comprehension-based account proposed in Roelofs (2004), self-monitoring uses the speech comprehension system but also involves a comparison process, which may be differently impaired. Moreover, the comprehension system is directly fed by the production system in self-monitoring but not in comprehending others. This makes self-monitoring and comprehending others differently sensitive to brain damage, which may explain the double dissociation between comprehension and self-monitoring ability in patients with aphasia. Finally, I explained why a domain-general ERN is compatible with comprehension-based monitoring, indicated that the ERN is related to error awareness, and discussed evidence against the claim that the ACC monitors for conflict. To conclude, in the light of the available evidence, the comprehension-based theory remains a viable account of self-monitoring in speaking.

## References

[1] Aarts, E., Roelofs, A., & Van Turennout, M. (2008). Anticipatory activity in anterior cingulate cortex can be independent of conflict and error likelihood. Journal of Neuroscience, 28, 4671–4678. DOI: 10.1523/JNEUROSCI.4400-07.2008 18448644 PMC6670453

[2] Blackmer, E. R., & Mitton, J. L. (1991). Theories of monitoring and the timing of repairs in spontaneous speech. Cognition, 39, 173–194. DOI: 10.1016/0010-0277(91)90052-6 1841032

[3] Botvinick, M. M., Braver, T. S., Barch, D. M., Carter, C. S., & Cohen, J. D. (2001). Conflict monitoring and cognitive control. Psychological Review, 108, 624–652. DOI: 10.1037/0033-295X.108.3.624 11488380

[4] Burle, B., Roger, C., Allain, S., Vidal, F., & Hasbroucq, T. (2008). Error negativity does not reflect conflict: A reappraisal of conflict monitoring and anterior cingulate activity. Journal of Cognitive Neuroscience, 20, 1637–1655. DOI: 10.1162/jocn.2008.20110 18345992

[5] Coles, M. G., Scheffers, M. K., & Holroyd, C. B. (2001). Why is there an ERN/Ne on correct trials? Response representations, stimulus-related components, and the theory of error-processing. Biological Psychology, 56, 173–189. DOI: 10.1016/S0301-0511(01)00076-X 11399349

[6] Dean, M. P., Della Sala, S., Beschin, N., & Cocchini, G. (2017). Anosognosia and self-correction of naming errors in aphasia. Aphasiology, 31, 725–740. DOI: 10.1080/02687038.2016.1239014

[7] Dronkers, N. F., & Baldo, J. V. (2009). Language: Aphasia In: Squire, L. R. (ed.), Encyclopedia of Neuroscience, 5, 343–348. Amsterdam: Elsevier DOI: 10.1016/B978-008045046-9.01876-3

[8] Freund, M., Gordon, B., & Nozari, N. (2016). Conflict-based regulation of control in language production. In: Papafragou, A., Grodner, D., Mirman, D., & Trueswell, J. C. (eds.), Proceedings of the 38th Annual Conference of the Cognitive Science Society, 1625–1630. Austin, TX: Cognitive Science Society.

[9] Gauvin, H. S., De Baene, W., Brass, M., & Hartsuiker, R. J. (2016). Conflict monitoring in speech processing: An fMRI study of error detection in speech production and perception. NeuroImage, 126, 96–105. DOI: 10.1016/j.neuroimage.2015.11.037 26608243

[10] Gratton, G., Coles, M. G., & Donchin, E. (1992). Optimizing the use of information: Strategic control of activation of responses. Journal of Experimental Psychology: General, 121, 480–506. DOI: 10.1037/0096-3445.121.4.480 1431740

[11] Guenther, F. H. (2016). Neural control of speech. Cambridge, MA: MIT Press.

[12] Hartsuiker, R. J., & Kolk, H. H. J., & Martensen, H. (2005). The division of labor between internal and external speech monitoring In: Hartsuiker, R. J., Bastiaanse, R., Postma, A., & Wijnen, F. (eds.), Phonological encoding and monitoring in normal and pathological speech, 187–205. Hove: Psychology Press DOI: 10.4324/9780203506196

[13] Hickok, G. (2012). Computational neuroanatomy of speech production. Nature Reviews Neuroscience, 13, 135–145. DOI: 10.1038/nrn3158 22218206 PMC5367153

[14] Holroyd, C. B., & Coles, M. G. H. (2002). The neural basis of human error processing: Reinforcement learning, dopamine, and the error-related negativity. Psychological Review, 109, 679–709. DOI: 10.1037/0033-295X.109.4.679 12374324

[15] Huettig, F., & Hartsuiker, R. J. (2010). Listening to yourself is like listening to others: External, but not internal, verbal self-monitoring is based on speech perception. Language and Cognitive Processes, 25, 347–374. DOI: 10.1080/01690960903046926

[16] Indefrey, P. (2011). The spatial and temporal signatures of word production components: A critical update. Frontiers in Psychology, 2 Article 255. DOI: 10.3389/fpsyg.2011.00255 PMC3191502 22016740

[17] Indefrey, P., & Cutler, A. (2004). Prelexical and lexical processing in listening In: Gazzaniga, M. (ed.), The cognitive neurosciences III, 759–774. Cambridge, MA: MIT Press.

[18] Indefrey, P., & Levelt, W. J. M. (2004). The spatial and temporal signatures of word production components. Cognition, 92, 101–144. DOI: 10.1016/j.cognition.2002.06.001 15037128

[19] Kröger, B. J., Crawford, E., Bekolay, T., & Eliasmith, C. (2016). Modeling interactions between speech production and perception: Speech error detection at semantic and phonological levels and the inner speech loop. Frontiers in Computational Neuroscience, 10 Article 51. DOI: 10.3389/fncom.2016.00051 PMC4885855 27303287

[20] Lackner, J. R., & Tuller, B. H. (1979). Role of efference monitoring in the detection of self-produced speech errors In: Cooper, W. E., & Walker, E. C. T. (eds.), Sentence processing, 281–294. Hillsdale, N.J.: Erlbaum.

[21] Lamers, M., & Roelofs, A. (2011). Attentional control adjustments in Stroop and Eriksen task performance can be independent of response conflict. Quarterly Journal of Experimental Psychology, 64, 1056–1081. DOI: 10.1080/17470218.2010.523792 21113864

[22] Levelt, W. J. M. (1983). Self-monitoring and self-repair in speech. Cognition, 14, 41–104. DOI: 10.1016/0010-0277(83)90026-4 6685011

[23] Levelt, W. J. M. (1989). Speaking: From intention to articulation. Cambridge, MA: MIT Press.

[24] Levelt, W. J. M., Roelofs, A., & Meyer, A. S. (1999). A theory of lexical access in speech production. Behavioral and Brain Sciences, 22, 1–38. DOI: 10.1017/S0140525X99001776 11301520

[25] Marshall, J., Robson, J., Pring, T., & Chiat, S. (1998). Why does monitoring fail in jargon aphasia? Comprehension, judgment, and therapy evidence. Brain and Language, 63, 79–107. DOI: 10.1006/brln.1997.1936 9642022

[26] Marshall, R. C., Rappaport, B. Z., & Garcia-Bunuel, L. (1985). Self-monitoring behavior in a case of severe auditory agnosia with aphasia. Brain and Language, 24, 297–313. DOI: 10.1016/0093-934X(85)90137-3 3978408

[27] Marslen-Wilson, W. (1990). Activation, competition, and frequency in lexical access In: Altmann, G. (ed.), Cognitive models of speech processing, 148–172. Cambridge: MIT Press.

[28] Motley, M. T., Camden, C. T., & Baars, B. J. (1982). Covert formulation and editing of anomalies in speech production: Evidence from experimentally elicited slips of the tongue. Journal of Verbal Learning and Verbal Behavior, 21, 578–594. DOI: 10.1016/S0022-5371(82)90791-5

[29] Nickels, L., & Howard, D. (1995). Phonological errors in aphasic naming: Comprehension, monitoring, and lexicality. Cortex, 31, 209–237. DOI: 10.1016/S0010-9452(13)80360-7 7555004

[30] Nozari, N., Dell, G. S., & Schwartz, M. F. (2011). Is comprehension necessary for error detection? A conflict-based account of monitoring in speech production. Cognitive Psychology, 63, 1–33. DOI: 10.1016/j.cogpsych.2011.05.001 21652015 PMC3135428

[31] Nozari, N., & Novick, J. (2017). Monitoring and control in language production. Current Directions in Psychological Science, 26, 403–410. DOI: 10.1177/0963721417702419

[32] Oppenheim, G. M., & Dell, G. S. (2008). Inner speech slips exhibit lexical bias, but not the phonemic similarity effect. Cognition, 106, 528–537. DOI: 10.1016/j.cognition.2007.02.006 17407776 PMC2435259

[33] Özdemir, R., Roelofs, A., & Levelt, W. J. M. (2007). Perceptual uniqueness point effects in monitoring internal speech. Cognition, 105, 457–465. DOI: 10.1016/j.cognition.2006.10.006 17156770

[34] Piai, V., Roelofs, A., Acheson, D. J., & Takashima, A. (2013). Attention for speaking: Domain-general control from the anterior cingulate cortex in spoken word production. Frontiers in Human Neuroscience, 7 Article 832. DOI: 10.3389/fnhum.2013.00832 PMC3856851 24368899

[35] Piai, V., Roelofs, A., Jensen, O., Schoffelen, J.-M., & Bonnefond, M. (2014). Distinct patterns of brain activity characterise lexical activation and competition in spoken word production. PLoS ONE, 9, e88674 DOI: 10.1371/journal.pone.0088674 24558410 PMC3928283

[36] Posner, M. I. (2012). Attention in a social world. Oxford, UK: Oxford University Press DOI: 10.1093/acprof:oso/9780199791217.001.0001

[37] Roelofs, A. (1997). The WEAVER model of word-form encoding in speech production. Cognition, 64, 249–284. DOI: 10.1016/S0010-0277(97)00027-9 9426503

[38] Roelofs, A. (2003). Goal-referenced selection of verbal action: Modeling attentional control in the Stroop task. Psychological Review, 110, 88–125. DOI: 10.1037/0033-295X.110.1.88 12529058

[39] Roelofs, A. (2004). Error biases in spoken word planning and monitoring by aphasic and nonaphasic speakers: Comment on Rapp and Goldrick (2000). Psychological Review, 111, 561–572. DOI: 10.1037/0033-295X.111.2.561 15065924

[40] Roelofs, A. (2005). Spoken word planning, comprehending, and self-monitoring: Evaluation of WEAVER++ In: Hartsuiker, R. J., Bastiaanse, R., Postma, A., & Wijnen, F. (eds.), Phonological encoding and monitoring in normal and pathological speech, 42–63. Hove, UK: Psychology Press.

[41] Roelofs, A. (2007). Attention and gaze control in picture naming, word reading, and word categorizing. Journal of Memory and Language, 57, 232–251. DOI: 10.1016/j.jml.2006.10.001

[42] Roelofs, A. (2008). Attention, gaze shifting, and dual-task interference from phonological encoding in spoken word planning. Journal of Experimental Psychology: Human Perception and Performance, 34, 1580–1598. DOI: 10.1037/a0012476 19045994

[43] Roelofs, A. (2014). A dorsal-pathway account of aphasic language production: The WEAVER++/ARC model. Cortex, 59, 33–48. DOI: 10.1016/j.cortex.2014.07.001 25128898

[44] Roelofs, A., & Hagoort, P. (2002). Control of language use: Cognitive modeling of the hemodynamics of Stroop task performance. Cognitive Brain Research, 15, 85–97. DOI: 10.1016/S0926-6410(02)00218-5 12433384

[45] Roelofs, A., & Piai, V. (2011). Attention demands of spoken word planning: A review. Frontiers in Psychology, 2 Article 307. DOI: 10.3389/fpsyg.2011.00307 PMC3209602 22069393

[46] Roelofs, A., Van Turennout, M., & Coles, M. G. H. (2006). Anterior cingulate cortex activity can be independent of response conflict in Stroop-like tasks. Proceedings of the National Academy of Sciences USA, 103, 13884–13889. DOI: 10.1073/pnas.0606265103 PMC1564252 16954195

[47] Salvucci, D., & Taatgen, N. A. (2011). The multitasking mind. Oxford University Press.

[48] Salvucci, D. D., & Taatgen, N. A. (2008). Threaded cognition: An integrated theory of concurrent multitasking. Psychological Review, 115, 101–130. DOI: 10.1037/0033-295X.115.1.101 18211187

[49] Shao, Z., Roelofs, A., Acheson, D. J., & Meyer, A. S. (2014). Electrophysiological evidence that inhibition supports lexical selection in picture naming. Brain Research, 1586, 130–142. DOI: 10.1016/j.brainres.2014.07.009 25219485

[50] Shitova, N. (2018). Electrophysiology of competition and adjustment in word and phrase production. Doctoral dissertation, Radboud University, Nijmegen.

[51] Vigliocco, G., & Hartsuiker, R. J. (2002). The interplay of meaning, sound, and syntax in sentence production. Psychological Bulletin, 128, 442–472. DOI: 10.1037/0033-2909.128.3.442 12002697

[52] Wessel, J. R. (2012). Error awareness and the error-related negativity: Evaluating the first decade of evidence. Frontiers in Human Neuroscience, 6 Article 88. DOI: 10.3389/fnhum.2012.00088 PMC3328124 22529791

[53] Wheeldon, L. R., & Levelt, W. J. M. (1995). Monitoring the time course of phonological encoding. Journal of Memory and Language, 34, 311–334. DOI: 10.1006/jmla.1995.1014

[54] Wheeldon, L. R., & Morgan, J. L. (2002). Phoneme monitoring in internal and external speech. Language and Cognitive Processes, 17, 503–535. DOI: 10.1080/01690960143000308

[55] Yeung, N., Botvinick, M. M., & Cohen, J. D. (2004). The neural basis of error detection: Conflict monitoring and the error-related negativity. Psychological Review, 111, 931–959. DOI: 10.1037/0033-295X.111.4.931 15482068

[56] Zheng, X., Roelofs, A., Farquhar, J., & Lemhöfer, K. (2018). Monitoring language selection errors in switching: Not all about conflict. PLoS ONE, 13, e0200397 DOI: 10.1371/journal.pone.0200397 30475803 PMC6261013

